# Ice, Natural or Artificial?

**Published:** 1889-09

**Authors:** W. M. Yandell

**Affiliations:** Health Officer, El Paso, Texas


DANIEL'S

### TEXAS MEDICAL JOURNAL.

A Representative Organ of the Medical Profession, and an Exponent of Rational Medicine; devoted to the Organization, Advancement and Elevation of the Profession in Texas.

PUBLISHED MONTHLY.—SUBSCRIPTION $2.00 A YEAR.

Vol. V. AUSTIN, SEPTEMBER, 1889. No. 3.

### ORIGINAL ARTICLES.

CONTRIBUTED EXCLUSIVELY TO THIS JOURNAL.

*The Articles in this Department are accepted and published with the understanding that we are not responsible for, nor do we indorse the views and opinions of the writers by so doing.*

For Daniel's Texas Medical Journal.]

### ICE, NATURAL OR ARTIFICIAL.

W. M. YANDELL, M. D., HEALTH OFFICER, EL PASO, TEXAS.

HAVING been called upon lately for an official opinion as to the relative purity of artificial and natural ice, I had occasion to study the question, and I submit to your readers the results of my investigations as my contribution towards correcting some popular, erroneous ideas.

In Pepper's System of Medicine, Vol. 1, page 185, article "Hygiene," by Dr. Jno. S. Billings, I find the following: "Ice is purer than the water from which it forms, but if cut on a foul pond, it will itself be foul, and the vitality of some microscopic organisms is not destroyed by their being frozen, as is shown by the fact that samples from the center of blocks of ice will inoculate sterilized infusions with the germs of putrefaction, precisely as the water of which the ice is composed, would have done before it was frozen. Disease has been traced to impure ice, and it may be that it is more frequently due to this cause than has heretofore been supposed; at all events it is well to bear the possibility in mind."

The following is from Parkes' Manual of Practical Hygiene, Vol. 1, page 21:

"Ice and snow often contain a good deal of suspended organic matter. Dr. Baker Edwards, of Montreal, found two grains per gallon in the shore ice and one grain per gallon in the river ice. Ice and snow may also be the means of conveying malarious poison to places at a distance."

In Vol. 2, same work, American Appendix, page 434, article on "Water" by Elwyn Waller, Ph. D., chemist to the health department of New York city, it is said: "The commonly received impression that water in freezing not only rejects all impurities, but that any germs, if frozen into it are necessarily killed, requires some correction. Water in freezing will enclose particles of organic or other matter which may be suspended in it, and almost any chemist can testify to the persistent vitality of many of the lower forms of organisms even after being imprisoned for a long time in blocks of ice, perhaps benumbed and dormant until released, but living. The general rule that a pond or river that is unfit as a water supply should not be used as a source of ice supply is a good one, but often disregarded."

H. E. Beebe, of Sidney, Ohio, in an article on "Influence of Ice and its Impurities in Disease", in the Annals of Hygiene, [Philadelphia] November, 1888, says:

"With their usual tenacious adherence to error and superstition, a majority of people hold to a firm belief in the theory, that water in freezing mechanically rids itself of all impurities and that ice must therefore, no matter from what source, be free from the dangers attributed to polluted water. But the fallacy of this has been proved by numerous experiments."

Speaking of bacteria, the same article says: "Freezing does not kill them, for Dr. Prudden has found twenty thousand living bacteria in a single cubic centimeter [about one-third of a teaspoonful] of ice. It is settled beyond dispute that the germs of typhoid and other fevers are carried to a large extent, if not almost wholly, in water, and these germs were the most abundant in the ice examined by Dr. Prudden. Nor does the danger lie only in small or stagnant bodies of water; the use of ice from Onondaga Lake was forbidden in Syracuse by the city board of health; the ice from the Hudson river below Albany has been proven dangerous from a similar cause. Localities not favored by the near presence of pure water, whence their supplies may be drawn at moderate expense, must depend upon ice made artificially from distilled water. This has other advantages besides safety over natural ice that must be hauled a considerable distance. It goes without saying, that in surgery no ice is to be brought in contact with the wounded flesh unless its absolute purity is beyond all question."

In the Sanitary Era, [New York and Chicago] May 15, 1887, report of James T. Gardiner, I find the following:

"There is a popular impression that water in freezing purifies itself; and for this reason many streams and ponds throughout the country are used for cutting ice for domestic supply that would be considered unfit for furnishing wholesome drinking water. The European records from which so much sanitary knowledge is drawn, are silent on this point, because ice has there been so little used, and because the ice supply of that region is largely brought from America and the far north. In the United States the attention of the medical profession seems not to have been directed to impure ice as a possible source of disease till 1879, when a mild but well marked epidemic occurred at Rye Beach, N. H., which was very thoroughly investigated by Dr. A. H. Nichols, of Boston, and the results fully reported to the State Board of Health of Massachusetts. The proof was unusually conclusive that the epidemic disturbance of the digestive organs was due to ice cut from a shallow pond containing a large amount of decomposing organic matter."

In the American Journal of Medical Sciences, January, 1878, Dr. C. Smart, Assistant Surgeon U. S. A., published the results of a most interesting investigation on "Mountain Fever and Malarious Water," in which he traced the origin of Mountain fever to the melting snow water in the Rocky mountain streams, and inferred that the germs of this typo-malarial fever were brought down from the atmosphere by the snow, and remaining frozen during the winter, passed into the streams with the melting snow in May, June and early July.

"In 1879, an epidemic of dysentery occurred in the village of Washington, Connecticut, which was investigated by Dr. Orlando Brown, of Litchfield, and Dr. J. R. Raymond, of Brooklyn. The ice used by the persons attacked was from a small and much polluted stream. The analysis of the ice itself showed it to be grossly contaminated. No other origin being found for the epidemic, which was limited to those who used the impure ice, the ice was pronounced to be the cause of the dysentery. The case was reported by the State Board of Health of Connecticut.

"In the report of the Connecticut Board for 1882, an isolated case is reported, where a private ice supply was cut from a pond which had received through a drain the dejecta of a typhoid patient. The report of the Connecticut State Board of Health for 1880, stated that several isolated cases of enteric trouble, and one death, were reported during that year, as due to free use of sewage-polluted ice."

In an editorial June 1, 1887, same journal, page 287, it is said:

"ARTIFICIAL ICE WORKS

Are highly successful in the South, in consequence of the expensive transportation and waste of Northern ice, and the perfect solidity and superiority in other ways, of the manufactured article. The choice of water for artificial ice secures greater purity, and when the ice manufacturers begin to realize that by a small expense for "plant" they can secure absolute purity, such as natural ice cannot have, without any practical addition to the cost of their product, they will be near the day when their ice will be the only kind thought fit for the table, and will be in universal demand. As people become educated to the importance of purity in water and the impossibility of being sure of it in natural ice, it will be more and more easy to obtain a fair market everywhere for the perfected article. There is far better reason to look for a general substitution of refined ice for crude, than there was a few years ago to suppose that brown sugar would disappear from the bins of grocers and the tables of the common people, and be replaced by the snow-white "loaf" that used to be a luxury of the rich. Not only is refined sugar now practically cheaper than brown, it has actually gone below the old price of brown sugar. There is far less reason why the like should not happen with refined ice. In both cases, it is a matter of enlarged operations, improved systems, and stimulated competition."

From the "Bullion," a mining paper of this city, edited by Prof. Chas. Longuemare, an expert chemist, July 23, 1889, I select the following description of the process employed in this city:

"ARTIFICIAL ICE.

"The manufacture of ice is an interesting process but little understood by the masses of the readers of newspapers, and in fact it is an operation that to fully understand requires some knowledge of natural philosophy and chemistry. The ice made by our modern refrigerators is due to the condensation, compression and expansion of ammonia. The process briefly consists of submitting ammonia, in the form of gas, to heavy pressure; this condensation developes heat. This heat is subsequently withdrawn from it by forcing it through a system of pipes that traverse water. The latter becomes charged with the heat; this operation condenses the gas into a liquid. The ammonia continues its course through pipes of larger dimensions. At this point, surrounded by a saturated solution of common salt, where it once more becomes possessed of its heat and thus restored to a gaseous state, and the water contained in the cans that are immerged in the salt brine parts with its heat, and becomes ice.

"The water used by this plant is secured from the El Paso water works. This water, as was previously stated in the Bullion, is more free of organic matter than that of the Mississippi or Ohio, taken respectively at St. Louis and Louisville. The water from the mains is run into a boiler of sufficient capacity, where it is evaporated, and in the form of steam continues its course to the condensers upon the third floor, in pipes. Here the distilled water is permitted to descend to the first floor, where it enters filters, where it is deodorized and purified as far as can be accomplished. The absolutely pure liquid is now forced into an hermetically closed reservoir, from whence it is drawn as required to fill the cans, that are, in fact, the moulds from which the absolutely pure ice is furnished to customers in the city, and elsewhere in the Southwest. We use the word absolutely pure, because, as has been observed by the reader, the water has been converted into vapor, leaving in the boiler its solid associations, and its conversion into steam has rendered it free from any organic matter or germs of life.

"The impression that ammonia at times enters into the manufactured ice is an error, for as can be seen in a previous portion of this article, the ammonia is in the form of vapor when near the congealing cans of water, contained in close fitting pipes, and if, perchance, a leak were to occur, the gas would be absorbed by the solution of salt and water, and could, under no circumstances, enter the receptacles containing the water intended for the manufactured ice, owing to the want of specific gravity in the escaping gas. In fact, the entrance of ammonia into the cans filled with water is absolutely impossible."

From a medical standpoint, doctors are not interested in the cost of ice, except that many patients might be debarred from its use by too great cost; but as citizens we are interested, in common with our fellow-citizens, in promoting manufactories in our town. Many small towns in Texas now have their ice plants, furnishing a luxury to those in health, and one of the most valuable of all curative agents in disease. With a greater demand, we may look for a cheapening in cost.

